# Three-Dimensional Laser Scanning for Geometry Documentation and Construction Management of Highway Tunnels during Excavation

**DOI:** 10.3390/s120811249

**Published:** 2012-08-14

**Authors:** Vassilis Gikas

**Affiliations:** School of Rural and Surveying Engineering, National Technical University of Athens, 9 I Polytechniou Str., Zographou, Athens 15780, Greece; E-Mail: vgikas@central.ntua.gr; Tel.: +30-210-772-3566; Fax: +30-210-772-2728

**Keywords:** laser scanning, LIDAR, tunnel excavation, total station, formwork, geometry documentation, construction management

## Abstract

Driven by progress in sensor technology, computer software and data processing capabilities, terrestrial laser scanning has recently proved a revolutionary technique for high accuracy, 3D mapping and documentation of physical scenarios and man-made structures. Particularly, this is of great importance in the underground space and tunnel construction environment as surveying engineering operations have a great impact on both technical and economic aspects of a project. This paper discusses the use and explores the potential of laser scanning technology to accurately track excavation and construction activities of highway tunnels. It provides a detailed overview of the static laser scanning method, its principles of operation and applications for tunnel construction operations. Also, it discusses the planning, execution, data processing and analysis phases of laser scanning activities, with emphasis given on geo-referencing, mesh model generation and cross-section extraction. Specific case studies are considered based on two construction sites in Greece. Particularly, the potential of the method is examined for checking the tunnel profile, producing volume computations and validating the smoothness/thickness of shotcrete layers at an excavation stage and during the completion of excavation support and primary lining. An additional example of the use of the method in the geometric documentation of the concrete lining formwork is examined and comparisons against dimensional tolerances are examined. Experimental comparisons and analyses of the laser scanning method against conventional surveying techniques are also considered.

## 1. Introduction

Today, tunnel construction projects are faced with more complex design specifications, tougher quality control standards and narrower construction times together with increasingly tighter budgets than ever before. In such a highly demanding working environment the role of surveying engineering becomes a critical aspect to the success of a tunnel project from initially planning through completion and final acceptance. In particular, surveying operations that aim at geometric documentation of a tunnel are concerned with all phases of the construction lifecycle (*i.e.*, excavation, completion of support measures, primary lining and tunnel commissioning). Besides, geometry documentation is of particular importance both to the contractor and the design engineers, and thus, it has a great impact on the technical as well as economic aspects of a project [1,2].

Traditionally, the surveying tasks relating to tunnel excavation operations have primarily relied on conventional surveying methods and to a lesser extent on photogrammetric techniques [3–7]. In the first category standard and reflectorless total stations are still extensively used to take profile measurements for design parameter verification and volume computations. More recently, specialized software applications have been made available that considerably automate field work and office calculations with a significant impact on operational efficiency and cost savings [8,9]. However, notwithstanding traditional surveying methods represent a flexible, precise and reliable solution to the problem they cannot provide a continuous representation of the tunnel surface. On the contrary, close range photogrammetry can provide image-based 3D models of a tunnel tube. Such models are produced using multiple images of an area suitably corrected for lens and perspective distortions [10]. For this purpose various processing techniques exist, ranging from stereoscopic vision of pair images to multi-convergent analysis supported by bundle adjustment. Nevertheless, although photogrammetric techniques offer a universal and relatively low-cost alternative, their use is less common in tunnel works due to the uneven wall surface and poor lighting conditions [11]. Also, despite the rapid advances on software tools that facilitate data collection and the processing cycle, stereoscopic plotting still requires expertise operators [10].

In recent years, the emergence of laser scanning technology has opened new perspectives for the recording and 3D reconstruction of a tunnel's wall at the various stages of a construction program. Terrestrial Laser Scanners (TLS) use the reflection of a focused laser beam from objects to compute their location in 3D space. High resolution TLS can deliver millions of point locations with high (several millimeters to centimeter) accuracy in a short time and, in many cases, under rough field conditions. Their use in the underground space environment is suited to a wide spectrum of application areas, ranging from civil engineering [1,12], to cave modeling [13] and archaeological documentation [10]. Section 3 provides a summary of current use of TLS in tunneling applications. From this review it is evident that despite the rapid expansion of TLS in tunneling operations, there still exists a lot of unexplored potential that, if adequately developed, would benefit greatly the tunneling industry. In effect, today, in spite of the many capabilities of TLS technology, the high cost of the equipment and the still developing algorithms for automatic data processing, along with the considerably long data acquisition times and complexity of data management might impose some practical constraints in certain cases.

The scope of this paper is twofold; firstly, to provide a detailed review on TLS technology, to outline its applications in tunnel construction and to discuss the practical and theoretical issues arising during collection and processing of the scan data; and secondly, to expound the potential of TLS in geometric documentation of tunnels under construction through comparisons with traditional surveying methods. More specifically, three case studies are discussed: (a) multiple scanning at the tunnel face for computing over-cuts/over-breaks and for surface characterization; (b) scanning the tunnel corridor for producing profile sections and volume calculations; and (c) scanning the metal arch formwork for verifying its structural dimensions against nominal geometry. The scan data discussed in this paper come from two construction sites in Greece: (a) a motorway tunnel which is currently under construction in Central Greece and; (b) a newly constructed tunnel of the Athens suburban railway system. The paper is divided into six sections. Following the introduction, the second section provides an overview to the TLS surveying method with emphasis given on static systems, whereas, the third section provides a review on the applications and potential of TLS in tunnelling. In the forth section, the scanning and profile generation process is discussed, followed by a detailed presentation of the use of the method through case study scenarios. Summary and key conclusions are presented in the final section.

## 2. The TLS Surveying Method

### 2.1. Overview of the Method

Terrestrial laser scanning enables the measurement and location of a large quantity of 3D points (known as the “point cloud”) in an automated manner and a very short time. In practical terms and in comparison to conventional surveying methods, the laser scanning technology offers a much higher point density data, an increased speed of data capture and the possibility for enhanced imagery and 3D visualization through specific processing and modeling tools. Also, compared to photogrammetric techniques, when complex and irregular objects of an uneven surface are to be documented the laser scanning method is usually the most appropriate option.

**Figure 1.**
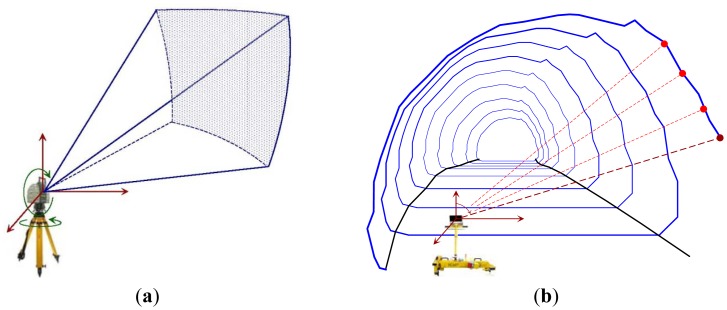
Working principle for the static (**a**) and kinematic; (**b**) terrestrial laser scanners.

Depending on the type of use, TLS can be operated either from a static position (mounted on a tripod) or from a dynamic platform (attached on a moving vehicle) [14,15]. In the first case, the TLS is used to produce a detailed map of the topographic features of the area around the static location that is occupied by the scanner, whereas in kinematic mode, it facilitates for conducting surveying and inventory maps of the corridor around the moving vehicle. The working principle of static TLS relies on repeated measurements of the slope range taken by an Electronic Distance Measurement (EDM) device at known angular intervals, which are defined at the horizontal and vertical planes passing through the origin of the EDM sensor. The outcome of this process is the spherical polar coordinates of the points in the field of view of the instrument in a local (topocentric) coordinate system. In contrast, in the case of kinematic laser scanning the device changes its position during data capturing. Therefore, a 3D point cloud emerges from the distance measurement, an angle measurement and the motion of the scanner [15]. Figure 1 offers a schematic view of the operation principle for both cases.

The operational principle of TLS is similar to that of a robotic total station. However, TLS do not include an optical sighting assembly, and therefore they do not have the ability to measure on very specific ground features. On the contrary, the measuring head of the instrument is set to carry out distance and angular measurements over a pre-defined angular range and field of view. This operation is performed at constant angular increments the size of which is typically set by the user. In addition to 3D polar coordinates, the laser scanners can measure the reflection intensity of the targets in sight. Reflection intensity (*i.e.*, the strength of returned laser beam) is greatly affected by the surface material, the angle of incidence and the distance between the scanner and the surveyed points. This information is critical in many applications, as it can be used to interpret predominant physical characteristics (such as roughness or material type) of the surface in question. Also, most laser scanners are equipped with a CCD camera, the location of which is precisely known with respect to the scanner's electro-optical center by means of accurate calibration. In the early days of TLS technology, the CCD unit was used for sketching purposes in order to allow the user to identify specific objects within the field of view of the instrument. Today, CCD cameras are also used for mesh-texturing imagery purposes [16–18].

### 2.2. Classification and Operating Principles of Static TLS

Static TLS systems can be classified in various ways. Among them, the most the widely adopted approach in the published literature relates to the distance measurement technique that a TLS system employ. According to this sorting approach three principal methods exist, known as, triangulation, interferometry and time-of-flight methods [19,20]. The first technique operates on the basis of optical triangulation; that is, a light source (*i.e.*, a single laser spot or a laser stripe) scans an object surface while its reflection is being recorded by one or more CCD cameras. The resulted distance is a function of the CCD inclination angle and the base-length defined between the CCD camera and the laser sensor. This method is accurate only over ranges of a few meters; and thus, is mainly used for industrial applications rather than in surveying engineering. In the second method, following the interferometric principle, an electromagnetic wave is split in two beams to produce an interference pattern that, if analyzed appropriately, can lead to the measured distance. However, this approach is suited only for short, ultra-precise (sub-millimeter) distance and displacement measurements [17].

Most TLS systems used for engineering geodesy applications employ the time-of-flight method [14,17]. In this case, two operating principles for distance measurement are in use: the pulsed time-of-flight (direct time-of-flight), and the phase difference (indirect time-of-flight) principle. In the first approach, the distance from a TLS sensor to a feature point is determined by measuring the time it takes a laser pulse to travel to it and getting back to the sensor. Subsequently, its 3D polar coordinates are computed using the measured distance together with the horizontal and vertical angles registered in the instrument. In contrast, in the case of phase-based scanners, the ranging principle resides on the phase difference obtained between the transmitted and the received (backscattered) signal from the scanned points. This technique applies to laser systems that emit a continuous string of a laser beam, in a way that, a series of successive range measurements is obtained [14]. Table 1 provides an overview of the mathematical formulae underlying the two operating principles.

From a practical perspective, the choice of a TLS depends on specific application needs that prescribe the technical characteristics of the scanner [19]. In general, the main features that characterize a TLS system are: the maximum observation distance, the scanning speed, the scanning resolution and the measurement quality (precision, accuracy and repeatability). As a matter of fact, these performance characteristics depend on the measurement method that a TLS employs. Thereby, the pulse-based scanners can measure long distances (in most cases <2,000 m); however, they operate at a reduced speed rate and a lower accuracy compared to phase-shift systems. In contrast, the latter can take measurements at high speed rates and high accuracies; however, the measuring range is limited (<100 m). In general, the pulse-based systems are well suited for surveying engineering and topographic works. Typical examples form the generation of 3D city models and the topographic surveying of industrial plants and large civil engineering structures. In contrast, the phase-based systems are mostly used for the detailed mapping of small-scale objects and physical scenarios, such as those encountered in reverse engineering problems and in the geometric documentation of cultural heritage objects.

## 3. Applications and Potential of TLS in Tunnel Construction Operations

Geodetic engineering operations relate to the entire lifecycle of a tunnel construction program. These can be classified in four distinct categories as follows: (a) horizontal and vertical geodetic control networks; (b) setting-out works and alignment of the excavation axis; (c) geodetic monitoring of ground displacements and tunnel convergence and; (d) detailed mapping of the tunnel corridor for geometry documentation, support of geological/geotechnical analysis and asset inventory purposes.

Geodetic control networks are required both on ground surface and the underground environment for subsequent surveying operations. Horizontal geodetic control on ground surface is usually undertaken by means of satellite geodesy (GPS observations), while vertical control points are established using precise leveling techniques [21]. Subsequently, ground surface networks are densified and stretched out to provide geodetic control for tunnel excavation. For this purpose open (zig-zag) or loop (polygon) traverses are established through the access portals and tunnel stairwells using conventional geodetic techniques and instrumentation. Similar methods are employed for staking-out the tunnel axis, the banquettes and vertical reference points. Also, integrated systems employing motorized laser and gyro-theodolite technologies are used to establish and maintain directional alignment of the excavation axis [4]. Laser scanners are generally not suited for setting-out operations. However, just a few of the latest models render simple setting-out functions, e.g., the Leica Geosystems, ScanStation 2. A comprehensive review of the principles and technologies employed for geodetic control and setting-out works in tunneling can be found in [1,15].

With regard to geometry documentation operations, TLS technology has proved to be a powerful mapping and quality control tool, together with well established surveying engineering methods. At an excavation stage, specific tasks include excavation profile control, under and over-cut detection and visualization as well as blast/drill pattern verification. During support measure operations, TLS can be employed for profile checks as well as for volume calculations and layer thickness determination of shotcrete (sprayed concrete), whereas at the final stages of construction, 3D laser scanning models can provide a thorough documentation of the tunnel surface [22–24]. TLS surveys can also be undertaken for asset inventory purposes. Such surveys lead to 3D models that feature comprehensive imaging and positional information and can be used for detailed inspection of the tunnel body and for documentation of its equipment. Also, they provide base data suitable for planning refurbishment projects [25]. Usually, for this type of surveys kinematic laser scanners are used, whereas, scanning of the excavation face and profile control checks is undertaken using static laser scanning systems.

The main advantages of TLS technology compared to conventional surveying techniques emerge in the high data volume and the potential that arises from the 3D modeling and visualization capabilities of the point cloud. Likewise, another asset of the laser scanning method is the reflection intensity information that is registered together with the positioning information. Recently, several examples exist in the published literature [26–28] that value this information in support of the geological/geotechnical analysis in tunneling. Mapping of the geological features (structure, texture and roughness), characterization of rockmass discontinuities (their location, spacing and orientation) and localization of potential leakage regions are goals of primary interest to the geologists and tunnel engineers. In order to obtain a dense map model of the excavation face, static laser scanners are used; preferably, phase-based systems that allow high density and high speed data acquisition (up to 10^6^ points per second).

Tunnel surface deformations reflect the evolution of the rock-mass behavior due to the excavation process. Traditionally, deformation analysis studies are based on displacement data obtained using conventional surveying and geotechnical techniques. These methods, notwithstanding can detect very fine (millimeter level) displacements; they measure the displacements at a limited number of points. In contrast, laser scanning is best suited for measurements over areas, but offers less precision. In fact, there are several studies in the literature [11,29–35] that examine the potential of TLS for displacement monitoring in tunneling. These studies suggest that TLS could possibly improve the understanding of rock-mass behavior as it allows for the mapping of displacements over an area rather than at specific points. However, the applicability and efficiency of the method greatly depends on the density and quality of the scan data, the processing technique used and the individual characteristics that define the physical phenomenon on a case by case basis.

**Figure 2.**
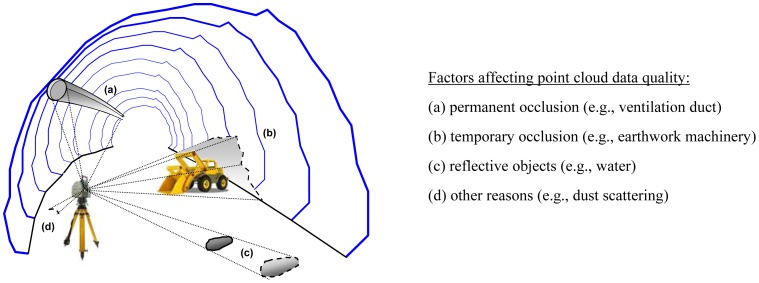
Factors affecting TLS data quality in tunnel construction surveys.

In general, modern TLS systems are robust enough to cope with the demanding operation conditions (such as dust and damp) found in the underground environment. Besides, TLS can operate effectively in darkness as the laser beam stands itself a light source. However, it is pointed out that despite the many advantages that induces the introduction of the laser scanning method in tunneling; its use implies a number of challenges and difficulties. In addition to certain limitations discussed already, the point cloud produced by TLS might not fully sample the scanning surface due to shadows relating to the relative geometry (viewing angles) between the instrument and the scanned section. Also, the presence of reflective objects (such as, equipment and water) in the field of view of the instrument can affect the recognition of targets (see Figure 2). In unstable rock conditions the scanning process might be furthered challenged. For example, it might be unsafe to set up the scanner close to an unsupported face, whereas long scanning sessions can lead unsafe conditions getting worse. As seen with operational and processing limitations that imply the use of the method, is inferred that laser scanning should not be regarded as an alternative to traditional measurement methods, but as a complement to well established surveying engineering practices. To this effect Table 2 attempts a classification of TLS usage in tunneling based on the description overview outlined in this section.

## 4. TLS Data Handling and Generation of the Tunnel Sections

### 4.1. Coordinate Systems in Tunnel Excavation

**Figure 3.**
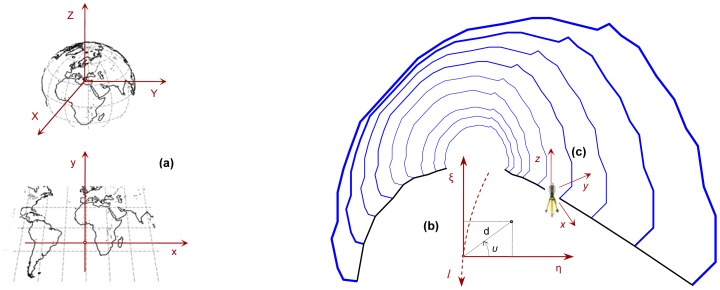
Coordinate reference systems used in tunnel construction: Geocentric, 3D Cartesian and associated projection coordinate system (**a**); linear reference system (**b**); and local coordinate system realized by instrument setup (**c**).

Figure 3 illustrates the various coordinate systems that are normally necessary for tunnel construction operations. In practice, three distinct, albeit mutually interrelated coordinate systems are used.

Firstly, a global 3D Cartesian coordinate system is adopted. This coordinate system forms the official geodetic frame of the project, used for geo-referencing purposes and for tying in the geodetic control network to neighboring construction or cadastral activities. For this purpose, a national geodetic reference system realized through an official map projection is usually selected. Secondly, a Linear Reference System (LRS) is employed to map the construction activities with respect to the tunnel centerline. The central axis or centerline of a tunnel is the combination of both horizontal and vertical alignments in 3D space. Therefore, such a coordinate system facilitates for producing plan, profile and cross-section views of the structure. As shown in Figure 3, a LRS can either be realized in the form of chainage (*l*), offset (*η*) and elevation (*ξ*) coordinates or, alternatively, in terms of chainage (*l*), oblique distance (*d*) and vertical angle (*υ*). Finally, engineering surveying and setting out operations necessitate a third coordinate system. In effect, the point locations surveyed from an instrument (total station or laser scanner) stationing are expressed at a local (topocentric) coordinate system the origin of which coincides with the electro-optical center of the instrument. In this coordinate system, the *z*-axis defines the direction of local vertical, the *y*-axis lies on the horizontal plane pointing towards an arbitrary chosen direction or towards the magnetic north, whereas the *x*-axis completes the right handed orthogonal coordinate system. Accurate and reliable coordinate transformations used for point cloud registration and for geo-referencing are of paramount importance in tunnel works, and thus, are examined closely in Section 4.2.

### 4.2. Planning the Scan Locations and Geo-Referencing the Point Cloud

As already stated, the scanning process is practically insusceptible to the underground lighting conditions. Therefore, the scanning operations are normally planned on the basis of area coverage as well as point cloud resolution and accuracy requirements with regard to survey specific goals and the technical characteristics of the scanner. Thereby, denser scan station setup spacing is required when a survey is intended to map the geological features of an area, as opposed to geometry documentation operations aiming at profile and volume computations. In the first case, a longitudinal spacing between instrument setups that equals the tunnel diameter would suffice most projects [27]. This spacing offset can be substantially increased if ordinary profile or volume computations are required. As a result, an increase in the spacing between instrument setups will lead in overly faster acquisition times together with lower data volumes.

No matter what the use of a TLS survey is meant for, adjacent scans are planned to overlap. In this regard, the points lying within the overlapped area are used to stitch (align) individual scans together, to form a continuous 3D scan image. For this purpose, a technique known as “fuzzy joint” is used to compute the optimal adjustment transformation parameters between neighboring scans [36,37]. The working principle of this technique resides on minimization of the root mean square (RMS) error of the residual distances involved for all points lying within the overlap zone. However, it should be noted that in tunnel works the method is prone to errors due to the longitudinal geometry, and thus, a substantial overlap in the surveyed areas between neighboring scans (corresponding at least 20% of a single scan data volume) is usually required. Alternatively, point cloud alignment can be achieved using only a limited number of pairs of feature points that appear in adjacent point clouds. Bolts, pipes or other clearly visible objects fixed on the tunnel surface can be used for this purpose. In this case an affine transformation computation is applied to compute the best transformation parameters between the coordinate systems that pertain to instrument setups of neighboring point clouds.

**Figure 4.**
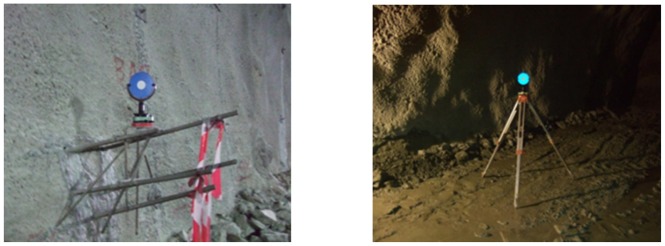
TLS spherical targets used for geo-referencing the point cloud.

Absolute positioning (geo-referencing) of the unified point cloud can be achieved if the coordinates of the feature points used to register adjacent point clouds are known in the global coordinate system employed for construction. This can be accomplished using special targets, the location of which is computed in the global coordinate system using a total station (see Figure 4). A simpler and faster, albeit less accurate technique resides on geo-referencing every point cloud acquired from a single scan shot independently from its neighboring point clouds. This technique works in two steps. Firstly, the point cloud data are shifted in regard to the global coordinate system by working out the location occupied by the scanner. Secondly, orientation of the scan data is accomplished using a known direction from the laser scanner position to a topographic prism that is visible in the point cloud. Dead reckoning of the laser scanner and prism locations is achieved by means of a total station.

### 4.3. Mesh Model Generation and Methods of Cross-Section Extraction

Two methods are widely used in tunneling for cross-section extraction from TLS data. The first method considers a subset data volume of the point cloud to form a thin, sliced, solid body, the sides of which are parallel to the vertical plane that defines the cross-section in question. In this case, the cross-section is realized by a poly-line string which results from projecting the point cloud data lying in the sliced body, on to the plane of the desired cross-section. Thereby, the final cross-section layout can be exported either in ASCII or CAD standard exchange format. A disadvantage associated with this technique relates to the extensive processing effort required in CAD software in order to generate the cross-sections from the selected points. However, it should be noted that this requirement does not represent a problem if specialized software for automatic generation of the cross-section polyline is used.

**Figure 5.**
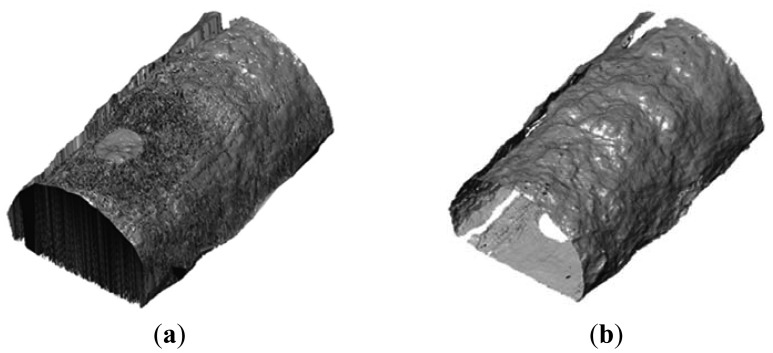
Example of a tunnel portion generated using a 2D mesh algorithm (**a**); and a successfully constructed 3D irregular mesh model (**b**); The problems associated with the use of a 2D mesh algorithm are evident at the top areas where the TIN triangles are actually “filling up” the space rather than forming a realistic representation of the tunnel surface. Also, at the sides of the tunnel the TIN (Triangulated Irregular Network) triangles are fictitiously elongated indicating failure in the 2D mesh model construction.

The second method presupposes the generation of a full 3D mesh model of the tunnel tube. Generally, the mesh generation refers to the practice of generating a polygonal or polyhedral mesh in the form of a 3D grid that approximates a geometric domain. Various algorithms exist for this purpose such as the polygon mesh and Delaunay triangulation [38]. Depending on specific application needs two types of mesh models can be considered. Firstly, regular type mesh models which constitute a mathematical representation of the surface in question, and secondly, irregular (or unstructured) type mesh models the geometric properties of which cannot be described by a regular mathematical surface. In tunnel surveying applications the regular mesh models is not an option. This is because the surface of a tunnel (especially at the early stages of construction), is so irregular that it cannot be approximated by a mathematical surface. Also, it should be noted that, the common 2D meshing algorithms usually employed for the generation of DTMs in road design, topographic surveying and GIS software packages cannot be used for the creation of a fully 3D irregular mesh model. This restriction stems from the fact that all ordinary 2D mesh algorithms assume a one to one correspondence between the scanned points and the reference plane (datum). Apparently, this is not the case in tunnel surveying as for every individual point fix on the projection surface (*i.e.*, the cross-section) correspond more than one point fixes on the tunnel wall. Today, the mesh modeling algorithms applied in TLS software have been specifically designed to face successfully this problem; and therefore, the mesh modeling method has prevailed in tunnel survey works. However, there exist situations where the processing analyst/engineer shall need to manually proceed to corrective actions to ensure quality assurance in the final product. Figure 5 shows an example of a successfully (3D) and an erroneously (2D) produced mesh model.

Conclusively, the 3D mesh modeling technique:

- allows slicing the TLS model along any desired direction to derive vertical cuts of the excavation model. This facilitates a close examination of the tunnel surface in 2D. Also, the detailed information associated with a mesh model offers the advantage of direct volume calculations of high accuracy. This is due to the fact that the method takes into account the tunnel profile information for the entire length between cross-sections,
- is by far more demanding in terms of computational time and data processing resources. This is due to the fact that the mesh model generation process can be an extremely computationally intensive task. For instance, it is noted that the generation of a mesh model for 1 km tunnel section might take up to several hours of processing time. However, it is noted that this time includes data handling operations and the effort required to divide the entire point cloud into smaller sections,
- has been observed to exhibit small errors of a local character, in the form of overlapping surfaces; and therefore, some special treatment is required during quality control.

## 5. Example Applications of TLS in Geometric Documentation of Tunnels

### 5.1. Description of Selected Sites and Instrumentation

The case study scenarios discussed in this paper come from two tunnel construction sites in Greece. The main application examples originate from a highway project (Tempi T1 tunnel). Besides, some individual examples from a railway construction program (N. Ikonio tunnel) are discussed on the side. The Tempi T1 tunnel is the first one of two twin-bore tunnels passing through the Kissavos Mountain in central Greece. It is located along the highway connecting Athens to Thessaloniki and is expected to be completed in 2013. The Tempi T1 tunnel is approximately 8.4 m high and 16.1 m wide with a total cross-section area of about 116 m^2^. It contains two lanes and a hard shoulder in each direction with cross passages every 300 m. Its length is approximately 1.9 km with an overburden ranging from about 10 m to 120 m. The tunnel passes through a variety of geologic formations mainly consisting of limestone of variable geotechnical characteristics, crystalline rocks and phyllites. The N. Ikonio tunnel is a single-track railway line located in the greater area of Athens. This tunnel serves as a suburban railway link-up of the Piraeus sea-port with the Thriassion railway freight terminal to the main railway network of the country. It is approximately 3.5 km long, 7.1 m high and 12.0 wide with a total cross-section area of about 66 m^2^. The tunnel is bypassing a military deployment and stretches close to a major natural gas pipeline supply network. The geologic setting in the excavation area consists of limestone and schists.

Scanning operations were undertaken using the Leica Geosystems ScanStation 2. This system is a high-speed, high accuracy pulse-based scanner suitable for a wide range of surveying engineering applications. It provides a maximum scan speed up to 50,000 points per second and features a horizontal and vertical field of view 360° × 270°. Its maximum measuring distance capability is 300 m at 90% reflectivity. Its range accuracy is 4 mm at 50 m observation distance. Data processing was accomplished using the Leica Geosystems Cyclone^®^ for viewing and geo-referencing purposes and the Technodigit 3DReshaper^®^ software for mesh modelling. In addition to ScanStation 2, a number of scanning shots at N. Ikonio tunnel were acquired using a somewhat older technology scanner, the Callidus CP-3200 laser scanner and Mensi GS-series^®^ processing software. Also, in order to provide comparisons between the TLS and conventional surveying techniques, the Leica Geosystems TCRM 1101 Plus total station and TMS PROwin^®^ software and the Trimble 5601DR total stations were used in the Tempi T1 and N. Ikonio construction sites respectively.

### 5.2. Tunnel Surface Documentation at the Excavation Face

**Figure 6.**
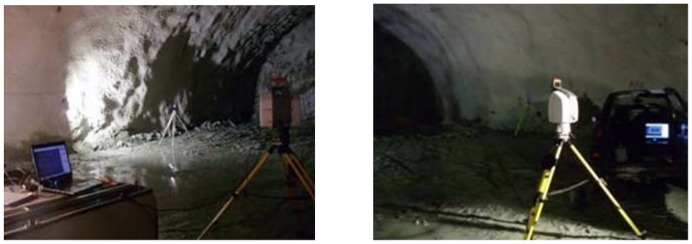
TLS setup during data acquisition. Depending on positional accuracy requirements four targets were used for geo-referencing the point cloud.

The laser scanning method has been adopted at Tempi T1 construction site for the purpose of documentation of the tunnel geometry and for geology characterization. This section provides several practical examples on the use of TLS technology for tunnel face documentation at two stages following the beginning of the excavation cycle; firstly, immediately after blasting and removal of the excavation material, and secondly, after a shotcrete layer has been applied [39]. Figure 6 shows the observation setup of the scanner and the sphere targets used for data geo-referencing. To ensure safe operation conditions and the best possible observation geometry, the scanner was setup approximately 10 m behind the tunnel face and within the limit of supported ground. Prior to the scan process the location of the TLS and the targets was computed by means of a total station. During data collection, the scanner recognizes automatically the targets in the point cloud, applies some fine scanning techniques and produces relative coordinates which are used for point cloud alignment of subsequent scans. In order to allow detailed mapping of the geologic conditions at the excavation face the angular spacing was defined equal to 0.001 radian increment. Also, digital images were captured at the same time with no need of additional lighting.

**Figure 7.**
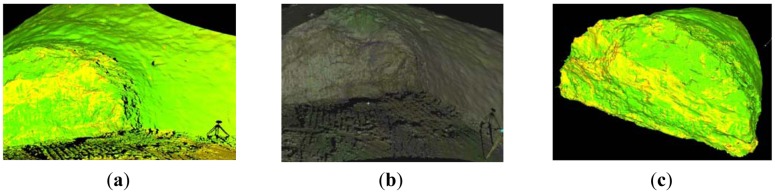
Raw TLS intensity data (**a**) and digital image; (**b**) obtained at tunnel Tempi T1 face immediately after blasting and excavation material removal; Laser intensity data of the same section, as seen from behind the tunnel face (**c**).

Figure 7 shows the raw laser intensity data (Figure 7(a)), and the corresponding digital image (Figure 7(b)) obtained at the tunnel face immediately after blasting. The limits of the excavation area and signs of rock perturbation due to the last advancement are clearly visible in the unsupported rock laser scanning image. Also, the intensity data from the laser scans, in many cases, can record efficiently the geological features of the excavation area–such as in Figure 7(c), where the change of rock type is evident. As stated already, laser scanning data can substantially enhance the geological/geotechnical documentation of the rock surface. However, this topic is not elaborated thereinafter in study and the interested reader is referred to consult Decker and Dove [26] and Fekete *et al.* [27].

**Figure 8.**
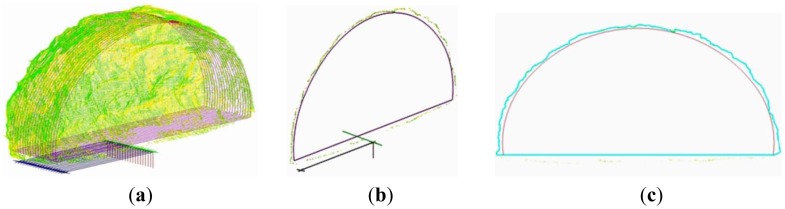
Cross-section generation at the excavation face based on raw point cloud data for the Tempi T1 tunnel.

**Figure 9.**
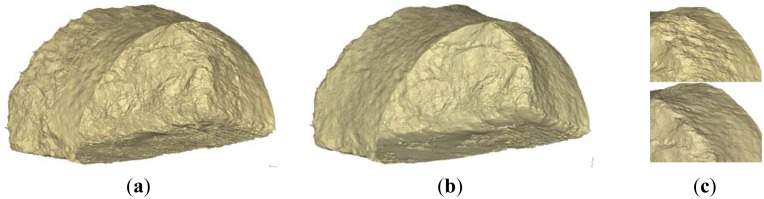
The mesh model computed for the laser scanning data shown in Figures 7 and 8. It shows the mesh model produced using the scan data after blasting (**a)**; after shotcrete layer has been applied (**b**); and a detailed view of the two cases (**c**).

Area and volume computations of the excavation material and shotcrete (sprayed concrete) are important for QA/QC purposes as well as for project accounting. As detailed in Section 4.3, this can be done, either directly on the point cloud data or using a 3D mesh model. Figure 8 shows an example of cross-section generation for computing over-breaks at the excavation face based on the raw point cloud data. For this purpose the typical cross-section is firstly overlaid on the raw (cleaned) scan data (Figure 8(a)). Then, a thin (<5 cm) slice of the point cloud (Figure 8(b)) is subtracted to construct a polyline that defines the excavated (actual) cross-section (Figure 8(c)). Thereby, the total over-break area (4.8 m^2^ or 4.14%) which corresponds to the chainage value of Figure 8(b) is computed by subtracting the typical cross-section area from the actual profile area. Cross-section information can also be produced employing a 3D mesh model. Figure 9 depicts the mesh model computed for the scan data shown in Figure 8.

**Figure 10.**
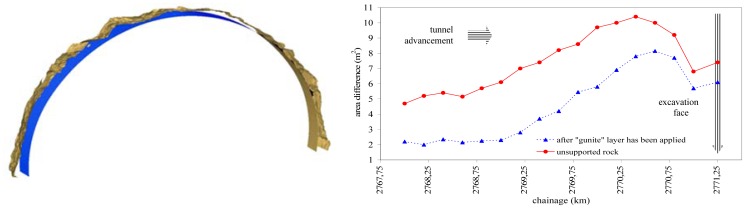
Subset slice of the mesh model overlaid on the design shell for the Tempi T1 tunnel. The plot on the right shows the cross-section area differences obtained between nominal and measured profiles after excavation and after shotcrete layer has been applied respectively.

As expected, the mesh model produced after a shotcrete layer has been applied (Figure 9(b)), is substantially smoother compared to the mesh model produced on unsupported ground (Figure 9(a)). Area and volume computations and their differences from the design values can be also produced using specialized software tools. As an example, Figure 10 shows a thin (1 m) slice of the mesh model of the excavated rock superposed upon the design shell. Moreover, Figure 10 presents the cross-section area computations obtained for an individual cycle length (*i.e.*, the excavation progress (advancement) achieved during a cycle of operations). More specifically, it shows the area differences obtained between nominal (design) and measured cross-sections for the case of unsupported rock and after a shotcrete layer has been applied. Such plots help to compute over-break material as well as shotcrete volume and thickness as a function of tunnel chainage with profound benefits to the project owner and the contractor.

### 5.3. Cross-Section and Volume Calculations during Support Measure Operations

After the first layer of shotcrete has been applied, the tunnel surface is measured for quality assurance purposes. The engineers use these data to obtain a detailed mapping of the construction sequence for two purposes: (a) to verify key features of tunnel geometry and optimize the procedure relating to concrete formwork; and (b) to provide project managers with high fidelity data to track construction activities. Traditionally, tunnel surface measurements are taken by means of a total station, either in a conventional way or using specialized software that automates field work and office computations. Alternatively, tunnel surveying can rely on laser scanning technology. This section provides comparison results of all three techniques based on sample data obtained from Tempi T1 and N. Ikonio case studies [40].

**Figure 11.**
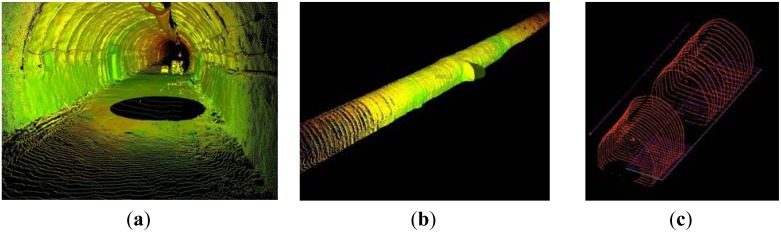
Raw point cloud data obtained for the Tempi T1 project (**a**), (**b**) and cross-section profiles extracted (**c**).

**Figure 12.**
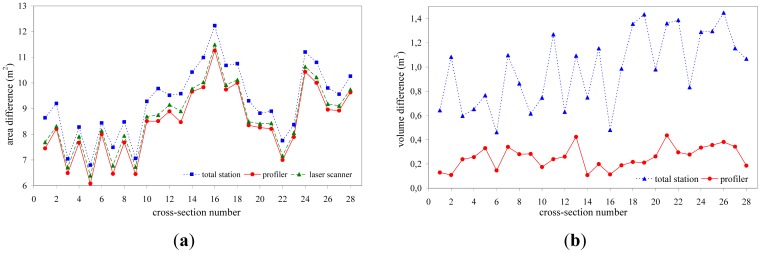
Cross-section area differences for the Tempi T1 tunnel case study obtained between nominal (theoretical) values and those measured for each observation technique (**a**); and over-break volume differences obtained between the manually and automatic total station surveys and those measured with the TLS method respectively (**b**).

For the case of the Tempi T1 tunnel the TCRM 1101 Plus spatial station was used to manually measure profiles at intervals 1.5 m over a tunnel portion 50 m long, collecting points every 1 m on each section. The same tunnel portion was measured using the TCRM 1101 Plus and TMS PROwin^®^ software at intervals of 1.5 m and every 1.0 m on each section. Finally, the same section was surveyed using the ScanStation 2 TLS from two locations spaced by 45 m (Figure 11). The limits of horizontal and vertical point spacing were defined so that, each cross-section is reconstructed using a minimum of 2,000 points. Figure 12 shows summary results for all three observation scenarios obtained at 28 cross-section locations. Figure 12(a) contains the profile area differences computed between the nominal values and those measured for each observation technique. From this plot it is evident that the over-break area increases from ∼7 m^2^ to ∼12 m^2^ and concludes to ∼10 m^2^. This fluctuation in over-cutting area represents a deviation between design and actually excavated volumes in the order of 6.4% to 11.0%, and might be due to variations in the geologic conditions or the blast process. However, the important thing to note from this diagram is that all measuring techniques exhibit the same variation pattern indicating consistency in the results obtained for each method. Also, as expected, the TLS method results in overall smaller differences due to an increased detail in the raw data. Furthermore, in order to examine the impact of the measuring technique in over-break volume computations, Figure 12(b) depicts two estimates. It shows the differences of inter-profile (*i.e.*, between consecutive sections) over-break volumes obtained between the manually and automatic total station surveys and those measured with the TLS method respectively. A thorough examination of this plot suggests that the observed differences in over-break volume computation can vary up to 1.4 m^3^ (∼6%) and 0.4 m^3^ (∼2%) per advancement meter, for the manual and automatic total station (profiler) surveys compared to the TLS method respectively. Notably, such differences, for a 2 km long tunnel, translate in an over estimate of concrete quantities of 2,800 m^3^ and 800 m^3^ depending which method is used.

**Figure 13.**
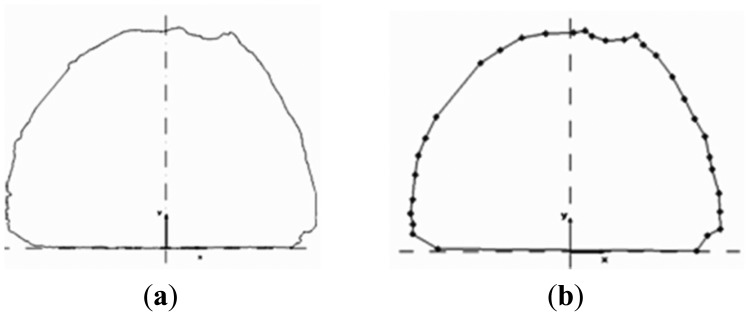
Example of cross-section extraction for the N. Ikonio tunnel based on the TLS (**a**) and total station (**b**) survey data. Note the lack of point information in the top left corner of plot (**b**) due to the ventilation duct system.

Analysis of the tunnel surface measurements obtained for the N. Ikonio case study leads into similar conclusions. A total number of 50 cross-sections were surveyed at a space interval 1.5 m and every 0.5 m on each section using the 5601DR total station. Also, the same portion was surveyed using the Callidus CP-3200 TLS unit. The sensor horizontal and vertical angular resolution was set to dHz = 0.25° and dV = 0.125°, which allowed the extraction of cross-sections containing 6,000 to 9,000 apex points. Figure 13 shows typical results of the analyses. From these plots the high resolution associated with the TLS method is immediately evident (Figure 13(a)). In contrast, for the case of the total station survey, the same cross-section contains only 32 points (Figure 13(b)). In fact, in these plots, the lack of information is more evident in the top left corner of the cross-section due to the ventilation duct system. Nevertheless, the differences observed in the over-break (over-cut) volume computations between the two methods are in the order of 5% and in agreement with the findings of the Tempi T1 case study.

Table 3 summarizes some key remarks of the use of TLS method in tunnel construction operations relating to geometry documentation and construction management.

### 5.4. Geometric Documentation of the Metal Arch Formwork

**Figure 14.**
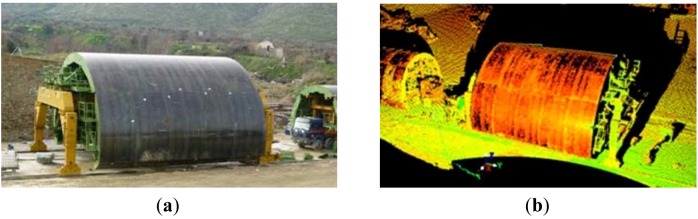
View of the formwork from the north portal abutment. The location of TLS stationing in the south abutment is denoted by a circle (**a**); The raw point cloud data recorded from the south portal abutment (**b**).

After tunnel excavation has been completed a permanent lining is installed. In the case of Tempi T1 tunnel, permanent support consists of cast in-situ concrete lining using formwork. The concrete lining process involves the use of a 12 m long metallic arch formwork travelling along the tunnel on temporary rails (Figure 14(a)). After the form is set into position a timber bulkhead is constructed at its open ends and concrete is pumped through openings (hatches) in the sides and top. Once lining is complete and concrete is sufficiently solidified, the form moves 12 m ahead and the same process is repeated.

The formwork comes into precast pieces and once is assembled is hard to manoeuvre (its weighs >150 t); and thus, is set to its final shape and location using specially designed hydraulic drive units. Therefore, in order to ensure final cross-section geometry and location (*i.e.*, position and orientation with respect to the tunnel axis), the formwork is repeatedly documented at the various stages of the process. Conventional geodetic surveys usually suffice for this purpose. However, in the case of the Tempi T1 construction site, in addition to routinely geometry checks relating to assembling and locating the form in space, it was deemed necessary to check the formwork for deviations in the nominal geometry of the precast metallic segments [41].

Such checks involved 3D geometric documentation of the form being in full expansion and comparisons against the design drawings. A combination of a high accuracy total station and laser scanning surveys was adopted for this purpose. The process involved the establishment of a loop transverse around the formwork. In order to obtain a clear view of the complete structure, a laser scanning survey was undertaken from two elevated locations established on the abutments by the tunnel portals (Figure 14(b)). The point clouds obtained from both instrument setups were co-registered into a single point cloud using as a common reference the locations of a number of special targets that appear in both field recordings. Field work was undertaken using Leica ScanStation 2 due to satisfying range accuracy (4 mm at 50 m). Data acquisition parameters were carefully selected, so that, scanning resolution (point cloud density) was better than 0.05 m.

**Figure 15.**
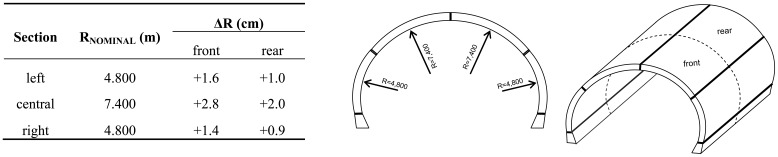
Differences in formwork geometry sub-sections obtained between nominal values and laser scanning surveys.

As shown in Figure 15, the nominal formwork cross-section geometry is represented by a series of connecting circumference cylindrical segments (notated as left, central and right). Therefore, data analysis involved partition of the point cloud into three pieces, each of them corresponding to a clearly defined geometric entity of the formwork, and fitting (using least squares) an appropriate solid figure (cylinder) to it. Besides, each of the three point clouds was further partitioned in two parts (front and rear), so that, detailed geometry statistics were produced for all sub-regions of the formwork. The outcome of the least squares adjustment for all sub-areas are shown in Figure 15. Analysis of these results for the front part indicates that, the arc radii of both sides of the formwork (left and right sections) deviate from their nominal values by less than 2.0 cm, whereas the top of the form (central section) exceeds the theoretical value by 2.8 cm—a difference, nearly twofold of the dimensional tolerance. Interestingly, from the same plot is evident that deviations from the nominal values are smaller at the rear of the formwork. These findings are of interest to the project owner and the contractor (e.g., 3 cm mean difference in the circumferential curvature radius for a 2 km long tunnel translates to 2,800 m^3^ extra (or less) lining material), and in certain cases (especially, for railway and metro tunnels) might be critical for the clearance traffic border line. The advantage of the laser scanning approach is that the uncertainties can be quantified and visualized, so that, they can be directly compared with the construction specifications for quality control purposes.

## 6. Summary and Conclusions

In this study, the potential and applications of laser scanning technology to collect high-fidelity data to support tunnel construction activities have been thoroughly examined. In particular, the primary focus of this work remains on the use of the method to accurately capture and analyze construction activities relating to geometry documentation and construction management of a tunnel project. The findings from its experimental use in two construction sites in Greece demonstrate the benefits and limitations of using TLS technology on a routine basis. The capability of laser scanning to provide a precise and accurate 3D mapping of the excavation site enables the construction sequence to be more transparent, faster and reliable compared to the data content available from conventional surveying approaches. Also, this capability benefits the tunnel engineers for a better understanding and controlling the various issues (geotechnical, geological, structural, *etc.*) arising during construction. From a contractual and project management perspective, TLS can prove valuable for better estimation of measures of quantities, planning of equipment resources and layout management.

It should be noted, however that, the quantitative findings resulted from the specific case study examples shown in this paper cannot be directly generalized. In fact, the performance (*i.e.*, level of feasibility and applicability) of laser scanning methodology can vary depending on individual site conditions, the excavation and support method used, as well as, pre-defined quality control specifications. Therefore, in order to make the most of laser scanning technology, the results of the method should be examined thoroughly on an individual project basis and adopt the capabilities it offers in complement with other surveying techniques.

## Figures and Tables

**Table 1.** Mathematical formulae used to compute the distance measurement (d) and distance resolution (Δd) for the pulse-based and phase-difference TLS computational methods.

**Parameter**	**Pulse-Based**	**Phase-Difference**		**Notation**
distance (d)	$c\cdot \frac{t}{2}$	$\frac{c}{2}\cdot T\cdot \left[N+\frac{\Delta \varphi }{2\pi }\right]$	t	flight time of light
			T	period of modulated signal
			c	speed of light in medium
resolution (Δd)	$c\cdot \frac{\Delta t}{2}$	$\frac{1}{4\pi }\cdot \frac{c}{f}\cdot \Delta \varphi$	Δφ	phase difference between received and transmitted signal
			N	number of waves
			f	frequency of modulated signal
			Δt	time resolution

**Table 2.** Classification overview of TLS applications in tunnelling operations.

**Applications of Terrestrial Laser Scanning in Tunnel Construction**			
geometric documentation	geological/geotechnical analysis	deformation monitoring	asset inventory surveys
excavation profile/volume control	geological features mapping	ground displacement and subsidence	3D “as-built” documentation
under & over-break calculation	rockmass discontinuities characterization	tunnel tube convergence	tunnel assets documentation
drill/blast pattern verification shotcrete layer thickness determination tunnel surface documentation	leakage regions mapping		

**Table 3.** Pros and cons of terrestrial laser scanning method for tunnel surface geometry documentation and construction management.

**TLS characterization in tunnelling operations**	

pros (+)	cons (-)
high spatial resolution (*suited for detailed QC checks*)	excessive processing work load
high production/efficiency rates	high cost of equipment (*compared to total station*)
3D modelling/visualization capabilities	heavy/voluminous equipment (*mainly older instruments*)
suitable for profile, area, volume check points	not-suitable for surveying individual points
useful data for other uses (*see* Table 2)	
